# Supplementary material for: A genome-scale metabolic model of Cupriavidus necator H16 integrated with TraDIS and transcriptomic data reveals metabolic insights for biotechnological applications
Source: PLoS Comput Biol. 2022 May 23;18(5):e1010106. doi: 10.1371/journal.pcbi.1010106 (PMC9166356; doi:10.1371/journal.pcbi.1010106)
Supplement: S6 Data — (Tables A-B): List of bacterial strains and plasmids and list of oligonucleotide primers used in this study. (DOCX) [file pcbi.1010106.s006.docx]

Table A: list of bacterial strains and plasmid used in this study.

| **Strain/plasmid** | **Details** | **Reference/source** |
| --- | --- | --- |
| ***E. coli*** | |  |
| DH5α | Standard laboratory cloning strain; genotype: F- *endA1 glnV44 thi-1 recA1 relA1 gyrA96 deoR nupG Φ80dlacZΔM15 Δ(lacZYA-argF)U169, hsdR17(rK- mK+), λ–* | [1] |
| S17-1 (λpir) | Standard laboratory conjugative strain; genotype: *recA pro hsdR RP4-2-Tc::Mu-Km::Tn7* | [2] |
| C2925 | Dam and Dcm methylation deficient strain; genotype: *ara-14 leuB6 fhuA31 lacY1 tsx78 glnV44 galK2 galT22 mcrA dcm-6 hisG4 rfbD1 R*(*zgb210::Tn10*) Tet^S^ *endA1 rspL136* (Str^R^) *dam13::Tn9* (Cam^R^) *xylA-5 mtl-1 thi-1 mcrB1 hsdR2* | New England Biolabs, Inc. |
| ***C. necator*** | |  |
| H16 | *C. necator* H16 wild type (DSM 428) | Leibniz Institute DSMZ-German Collection of Micro-organisms and Cell Cultures, Braunschweig, Germany |
| ΔA0792 | Mutant strain carrying the in-frame deletion of the *H16_A0792* (*pheA*) gene, encoding a prephenate dehydratase | This study |
| ΔA3038 | Mutant strain carrying the in-frame deletion of the *H16_A3038* (*nadA*) gene, encoding a quinolinate synthase | This study |
| ΔA3084 | Mutant strain carrying the in-frame deletion of the *H16_A3084* (*panB*) gene, encoding a 3-methyl-2-oxobutanoate hydroxymethyltransferase | This study |
| ΔA3165 | Mutant strain carrying the in-frame deletion of the *H16_A3165* (*ubiC*) gene, encoding a chorismate pyruvate-lyase | This study |
| ΔA3408 | Mutant strain carrying the in-frame deletion of the *H16_A3408* (*hisE*) gene, encoding a phosphoribosyl-ATP pyrophosphatase | This study |
| ΔA3434 | Mutant strain carrying the in-frame deletion of the *H16_A3434* (*aroB*) gene, encoding a 3-dehydroquinate synthase | This study |
| **Plasmids** |  |  |
| pMTL70115 | Suicide transposon delivery vector carrying the *tnpA* transposase-encoding gene and the miniTn5*::tetA* transposon | [This](#_ENREF_30) study |
| pMTL70621-SacB | Suicide modular vector used for deletion of genes in *C. necator*; carries the antibiotic resistance gene *tetA* and the *sacB* gene as a counter-selection marker | [3] |
| pMTL70621-SacB::ΔA0792 | pMTL70621-SacB derivative for deletion of *H16_A0792* (*pheA*) | This study |
| pMTL70621-SacB::ΔA3038 | pMTL70621-SacB derivative for deletion of *H16_A3038* (nad*A*) | This study |
| pMTL70621-SacB::ΔA3084 | pMTL70621-SacB derivative for deletion of *H16_A3084* (*panB*) | This study |
| pMTL70621-SacB::ΔA3165 | pMTL70621-SacB derivative for deletion of *H16_A3165* (*ubiC*) | This study |
| pMTL70621-SacB::ΔA3408 | pMTL70621-SacB derivative for deletion of *H16_A3408* (*hisE*) | This study |
| pMTL70621-SacB::ΔA3434 | pMTL70621-SacB derivative for deletion of *H16_A3434* (*aroB*) | This study |

Table B: list of oligonucleotide primers used in this study.

| **Primer** | **5’ to 3’ sequence** | **Details** |
| --- | --- | --- |
| MCSTn5_FOR | GCTTCCCGGGGATCAT | Primers used to verify insertion of the miniTn5*::tetA* transposon in the *C. necator* H16 genome |
| Tn5_NCOseq 3_REV | GTGGCGGGACCAGTGA |  |
| A0792_U_Fwd | tttatcaggaaacagctatgaccgcggccgcGTTCCAGGACATTCCCGATATC | Primers used to amplify the *H16_A0792* upstream region. Sequences in capital letters anneal on the *C. necator* H16 genome. Primer A0792_U_Fwd was also used to screen for H16 *ΔH16_A0792* mutants, in combination with A0792_D_Rev |
| A0792_U_Rev | ggctgccttacttTGTCATTTTGTATGAATCCGG |  |
| A0792_D_Fwd | atacaaaatgacaAAGTAAGGCAGCCAGGCAAG | Primers used to amplify the *H16_A0792* downstream region. Sequences in capital letters anneal on the *C. necator* H16 genome. Primer A0792_D_Rev was also used to screen for H16 *ΔH16_A0792* mutants, in combination with A0792_U_Fwd |
| A0792_D_Rev | tgccaagcttgcatgtctgcaggcctcgagACACCATCAGGTTCGGGTAG |  |
| A3038_U_Fwd | tttatcaggaaacagctatgaccgcggccgCTGATCATTGGTGGCGAAGAG | Primers used to amplify the *H16_A3038* upstream region. Sequences in capital letters anneal on the *C. necator* H16 genome. Primer A3038_U_Fwd was also used to screen for H16 *ΔH16_A3038* mutants, in combination with A3038_D_Rev |
| A3038_U_rev | ttcacgctcatgcGGTCATCTCCGGTACTCCTC |  |
| A3038_D_Fwd | accggagatgaccGCATGAGCGTGAATTCGATTTTC | Primers used to amplify the *H16_A3038* downstream region. Sequences in capital letters anneal on the *C. necator* H16 genome. Primer A3038_D_Rev was also used to screen for H16 *ΔH16_A3038* mutants, in combination with A3038_U_Fwd |
| A3038_D_Rev | tgccaagcttgcatgtctgcaggcctcgagATTGATCCTGACCGCATCCTG |  |
| A3084_U_Fwd | tttatcaggaaacagctatgaccgcggccGCCATGCTCGATCACCTGCG | Primers used to amplify the *H16_A3084* upstream region. Sequences in capital letters anneal on the *C. necator* H16 genome. Primer A3084_U_Fwd was also used to screen for H16 *ΔH16_A3084* mutants, in combination with A3084_D_Rev |
| A3084_U_Rev | caccggctcaggcGCCCATATAGGACGGCTTTGC |  |
| A3084_D_Fwd | gtcctatatgggcGCCTGAGCCGGTGGAGATCG | Primers used to amplify the *H16_A3084* downstream region. Sequences in capital letters anneal on the *C. necator* H16 genome. Primer A3084_D_Rev was also used to screen for H16 *ΔH16_A3084* mutants, in combination with A3084_U_Fwd |
| A3084_D_Rev | tgccaagcttgcatgtctgcaggcctcgaGTGGTCATGGGCAACTGAGG |  |
| A3165_U_Fwd | tttatcaggaaacagctatgaccgcggccgCGACCACGTGCTGGTGATGAGC | Primers used to amplify the *H16_A3165* upstream region. Sequences in capital letters anneal on the *C. necator* H16 genome. Primer A3165_U_Fwd was also used to screen for H16 *ΔH16_A3165* mutants, in combination with A3165_D_Rev |
| A3165_U_Rev | tgcagtttcatctGCTCATGCCGGGCCTGTGCC |  |
| A3165_D_Fwd | gcccggcatgagcAGATGAAACTGCAGGGTCGG | Primers used to amplify the *H16_A3165* downstream region. Sequences in capital letters anneal on the *C. necator* H16 genome. Primer A3165_D_Rev was also used to screen for H16 *ΔH16_A3165* mutants, in combination with A3165_U_Fwd |
| A3165_D_Rev | tgccaagcttgcatgtctgcaggcctcgaGCAGTTCAGAGCGACATGCC |  |
| A3408_U_Fwd | tttatcaggaaacagctatgaccgcggccgCTGGATGCGGTCGAATGGGC | Primers used to amplify the *H16_A3408* upstream region. Sequences in capital letters anneal on the *C. necator* H16 genome. Primer A3408_U_Fwd was also used to screen for H16 *ΔH16_A3408* mutants, in combination with A3408_D_Rev |
| A3408_U_Rev | ccagctagtccttGTCGCTCATGGCTTGGTGTAG |  |
| A3408_D_Fwd | agccatgagcgacAAGGACTAGCTGGCGCAGGC | Primers used to amplify the *H16_A3408* downstream region. Sequences in capital letters anneal on the *C. necator* H16 genome. Primer A3408_D_Rev was also used to screen for H16 *ΔH16_A3408* mutants, in combination with A3408_U_Fwd |
| A3408_D_Rev | tgccaagcttgcatgtctgcaggcctcgagCTAGCAAGCAGCTCCCCTTC |  |
| A3434_U_Fwd | tttatcaggaaacagctatgaccgcggccgcGAAGATGATGCAGTTCCC | Primers used to amplify the *H16_A3434* upstream region. Sequences in capital letters anneal on the *C. necator* H16 genome. Primer A3434_U_Fwd was also used to screen for H16 *ΔH16_A3434* mutants, in combination with A3434_D_Rev |
| A3434_U_Rev | cgatcggtcaggcAATCATGGATTGGGGTCC |  |
| A3434_D_Fwd | ccaatccatgattGCCTGACCGATCGGTTATG | Primers used to amplify the *H16_A3434* downstream region. Sequences in capital letters anneal on the *C. necator* H16 genome. Primer A3434_D_Rev was also used to screen for H16 *ΔH16_A3434* mutants, in combination with A3434_U_Fwd |
| A3434_D_Rev | tgccaagcttgcatgtctgcaggcctcGAGCTGCTCCAGCGTCAG |  |
| A0792_int_FOR | GAGGTCGGTGAGGTCAAGAA | Primers annealing within the *H16_A0792* gene, used to confirm H16 *Δ H16_A0792* mutants |
| A0792_int_REV | AGGTACCGACAGGATCATCG |  |
| A3038_int_FOR | ATCGATCAAGACCGTCGAGT | Primers annealing within the *H16_A3038* gene, used to confirm H16 *Δ H16_A3038* mutants |
| A3038_int_REV | TGGATATAGCTGCCCAGGTG |  |
| A3084_int_FOR | CCATGAGCTACCTCCTCGAT | Primers annealing within the *H16_A3084* gene, used to confirm H16 *Δ H16_A3084* mutants |
| A3084_int_REV | GTCAGCGATTGCGTGATCT |  |
| A3165_int_FOR | ACCTGGCCTTTGATGCAG | Primers annealing within the *H16_A3165* gene, used to confirm H16 *Δ H16_A3165* mutants |
| A3165_int_REV | TAGAGGGGCTTCGGTCTGTA |  |
| A3408_int_FOR | GACAACCAACTCAGCAGCAA | Primers annealing within the *H16_A3408* gene, used to confirm H16 *Δ H16_A3408* mutants |
| A3408_int_REV | AAGTTGGCCAGCAATACCAT |  |
| A3434_int_FOR | GCAGCTACCCCATCCATATC | Primers annealing within the *H16_A3434* gene, used to confirm H16 *Δ H16_A3434* mutants |
| A3434_int_REV | GTGTGGCCGAAATTGAGAAT |  |

**References**

1. Grant SG, Jessee J, Bloom FR, Hanahan D. Differential plasmid rescue from transgenic mouse DNAs into Escherichia coli methylation-restriction mutants. Proc Natl Acad Sci U S A. 1990;87(12):4645-9.

2. Herrero M, de Lorenzo V, Timmis KN. Transposon vectors containing non-antibiotic resistance selection markers for cloning and stable chromosomal insertion of foreign genes in gram-negative bacteria. J Bacteriol. 1990;172(11):6557-67.

3. Ehsaan M, Baker J, Kovacs K, Malys N, Minton NP. The pMTL70000 modular, plasmid vector series for strain engineering in Cupriavidus necator H16. J Microbiol Methods. 2021;189:106323.
