# Supplementary material for: A genome-scale metabolic model of Cupriavidus necator H16 integrated with TraDIS and transcriptomic data reveals metabolic insights for biotechnological applications
Source: PLoS Comput Biol. 2022 May 23;18(5):e1010106. doi: 10.1371/journal.pcbi.1010106 (PMC9166356; doi:10.1371/journal.pcbi.1010106)
Supplement: Attachment — Submitted filename: ResponseLetter_PCOMPBIOL-D-21-02005R1.pdf [file pcbi.1010106.s007.pdf]

Synthetic Biology Research Centre
University of Nottingham BioDiscovery Institute
The University of Nottingham
University Park
Nottingham NG7 2RD
t: +44 (0)115 95 15151
e: nicole.pearcy@nottingham.ac.uk
www.nottingham.ac.uk

PLOS Computational Biology
Carlyle House
Carlyle Road
Cambridge, CB4 3DN
United Kingdom

21st February 2022

Dear Prof. Maranas,

Please find attached our revised manuscript titled "A genome-scale metabolic model of *Cupriavidus necator* H16 integrated with TraDIS and transcriptomics data reveals metabolic insights for biotechnological applications". We have significantly revised the manuscript taking all comments made by the reviewers into consideration. We would like to thank both reviewers for their helpful comments and suggestions that have helped us strengthen our manuscript. The main changes to the manuscript are of follows:

1. A detailed comparison of *i*CN1361 to the recently updated RehMBEL1391 GSM of *C. necator* H16.
2. A comparison of recently published Tn-Seq data to our TraDIS and *i*CN1361 gene essentiality classifications.
3. Improved our approach for classifying genes as essential or non-essential using our TraDIS data.

A point-by-point response to the reviewers' comments is provided below. The original questions from the reviewers are in blue, whilst our responses are in black. The line numbers stated refer to the clean version of the manuscript. We hope these responses make our manuscript suitable for acceptance in PLOS Computational Biology and we look forward to hearing from you.

With best wishes, the authors

Dr Nicole Pearcy
Research Fellow

Dr Marco Garavaglia
Research Fellow

Reviewer #1:

Major comments

Regarding the model reconstruction, it would be more helpful to the readers if the authors can provide some comparison with the published models. For example, iCN1361 has 1292 reactions, while the number of reactions in the first RehMBEL1391 model is 1391 and the recent RehMBEL1391 model is 1360. Can the authors add some explanation on why certain reactions are added/removed? A good example is in the readme of the updated iCN1361 repos that include a list of changes made compared to the previous model (https://github.com/m-jahn/genome-scale-models).

We agree that a comparison between the latest version of RehMBEL1391 will be useful to readers and would like to thank the reviewer for highlighting this issue. In the revised manuscript we have included an additional section that includes a table highlighting the differences between the models' properties, which includes some of the main results from the MEMOTE reports from both models. Whilst RehMBEL1391 includes a significantly higher number of total reactions compared to *i*CN1361, the number of internal reactions, and importantly gene coverage, is actually very similar. RehMBEL1391 includes 384 transporters, however, only 17 of these have a gene association. In *i*CN1361, on the other hand, we only include transporters for substrates that are supported by experimental evidence. Other notable differences in reactions are caused by differences in model construction pipelines, including different definitions of fatty acid and lipopolysaccharides biosynthesis, as well as different information about enzyme specificity across databases (i.e., KEGG vs. BioCyc). We provide detailed information in our Supplementary Jupyter Notebook ('Comparisons_RehMBEL13691.ipynb', please also see below). Moreover, using the MEMOTE report (general results shown in Table 1, also see below) we could show that *i*CN1361 has 100% stoichiometric consistency, reactions are mass balanced, free from erroneous energy generating cycles and contains no unconserved metabolites, whereas stoichiometric and mass inconsistencies remain a problem in RehMBEL1391.

Additionally, we have carried out some comparisons of the models' predictive capabilities, including growth predictions, comparisons to ^{13}C metabolic flux analysis and gene essentiality phenotypes. Despite the differences in the reactions (mentioned above), we found that the growth predictions and central carbon metabolism fluxes are relatively similar between *i*CN1361 and RehMBEL1391 for growth on fructose. Notably, however, the RehMBEL1361 model appears to have an unrealistically high phosphate (and proton) uptake rate, which is likely a consequence of the stoichiometric imbalances. The overall accuracy of the gene essentiality predictions was also similar between the two models; however, our model scored a higher recall and precision, due to our model having fewer false positive predictions. These results are summarised on lines 209 - lines 227 (please also see below) but are provided in detail in an additional supplementary Jupyter Notebook 'Comparisons_RehMBEL1391.ipynb'.

Finally, please note that whilst we agree that listing the individual changes made to newer versions of the model is very informative to the readers, it is not feasible in our case as our model was independently constructed (rather than an extension/corrected version of an original model), and therefore would require mapping the 1292 reactions in *i*CN1361 to the RehMBEL1391 model to be able to list the differences. Whilst the updated model of RehMBEL1391 contains considerably larger number of identifiers (such as KEGG, BioCyc, etc.), there is still over 50% of the reactions that would require manual mapping. The approach we used to construct the model, however, uses the BioCyc flatfiles for *C. necator* H16 (v. 21.0) to generate a draft model. Any reactions that are removed or manually added/corrected to construct the final version *i*CN1361 are stored in the ScrumPy model files (e.g., removed reactions from the draft model are included in the file 'Unwanted.py' and manually added reactions are added to the model via the file 'ExtraReacs.spy'), and thus is informative for future users and developers of the model. To illustrate the model construction pipeline clearer, we now provide an additional supplementary figure on the GitHub page, to direct readers where this information is stored and how they can continue the development of the model using ScrumPy. The edits to the manuscript (Introduction, Results and Discussion sections) are provided below, as well as the results that summarise the model analysis comparisons.

Line 135 – line 140 (Introduction):
Notably, the authors of (25) have made some significant improvements to the original RehMBEL1361 in their recent publication, including the addition of identifiers for a subset of metabolites and reactions. Stoichiometric and mass inconsistencies, however, remain in this updated model, which are challenging to correct without the manual curation of metabolite and reaction information due to the incomplete coverage of database-linked identifiers.

Line 209 – line 227 (Results):
Comparison to RehMBEL1391

We have carried out a comparison of the network properties of *i*CN1361 to the recently updated version of RehMBEL1391 GSM of *C. necator* H16 (Table 1). It should be noted that the significantly higher number of reactions in the recently updated version of RehMBEL1391 is mostly caused by a considerable larger number of reactions associated with transport. Only 17 of these transport reactions have been assigned a gene association, and thus it is unclear whether *C. necator* H16 is able to carry out the function. In *i*CN1361, we instead include only transporters where experimental evidence confirms the function is present. In all other categories listed in Table 1, our model *i*CN1361 scores higher than the updated version of RehMBEL1391. The MEMOTE report for RehMBEL1391 shows that, in strong contrast to *i*CN1361, stoichiometric inconsistencies and reaction imbalances remain in the model despite the significant improvements. A more detailed comparison of the two models, including a comparison of their predictive capabilities, can be found on our GitHub

repository:
https://github.com/SBRCNottingham/CnecatorGSM/blob/main/JupyterNotebooks/Comparisons_RehMBEL1391.ipynb.

Table 1: Network property comparisons between *i*CN1361 and RehMBEL1391

Property	*i*CN1361	RehMBEL1391 (25)
Number of reactions	1292	1538
Number of transporters	98	384
Number of internal reactions	1194	1154
Number of metabolites (cytosol)	1263	1172
Number of genes	1361	1345
Reactions with gpr*	1110	1051
Blocked reactions	443	593
Functional reactions	793	738
Balanced reactions (%)	99.67	49.23
Erroneous energy-generating cycles	0	0
Unconserved metabolites	0	235
Stoichiometric consistency	yes	no

*GPR: Gene-protein-reaction relationships (see the Methods section for a detailed explanation)

Line 833 – line 841 (Discussion):

A major challenge in GSM development and analysis, however, is the readability and reusability of models. Often GSMs, including the original GSM of *C. necator* H16 (9), are constructed using non-systematic identifiers for metabolites and reactions, which considerably complicates the application of analysis packages, interpretation of results and further improvements of the model. Although a huge effort was recently made to improve the model readability of RehMBEL1391 (25), the lack of identifiers and chemical formulae for a subset of metabolites, makes it difficult to perform theoretical validation, such as stoichiometric and mass balance checks.

Summary of the comparisons in 'Comparisons_RehMBEL1391.ipynb'

GitHub link:
https://github.com/SBRCNottingham/CnecatorGSM/blob/main/JupyterNotebooks/Comparisons_RehMBEL1391.ipynb

In the following we list some observed differences of the reactions in *i*CN1361 compared to RehMBEL1391:

- Transport reactions: In our model we added transporters only for minimal media substrates and for carbon sources known experimentally to enable growth. Annotating transport reactions is much more challenging, so we only added a transporter for which experimental evidence is available. Transporters were also added for metabolites commonly produced by bacteria, in the same way as (Weaver 2014). The transporters in RehMBEL1391 were defined in the original model, but the majority lack a gene annotation, so it is unclear whether the transporter is present.

- Cell wall metabolism: In our model we include the full pathway for each fatty acid included in the model, and for the lipopolysaccharides (LPS), whereas RehMBEL1391 uses a lumped reaction of these pathways. Using the full pathway is useful when integrating transcriptomics data and/or proteomics data, otherwise the genes will need to be weighted according to the individual reactions involved in the lumped version.

- Biomass equation: The way in which the biomass reaction is defined is slightly different in the two models. In *i*CN1361 the biomass reaction includes all individual biomass components (i.e., alanine + aspartate + dATP + dGTP + … → Biomass), whereas in RehBMEL1391, it is composed of separate reactions for each macromolecule (i.e., protein + DNA + … → Biomass). However, these are just different ways of formulating the biomass equation and do not affect the model results.

- Enzyme specificity: *i*CN1361 was constructed using the BioCyc database, whereas the RehMBEL1391 has assigned gene annotations using KEGG and UniProt. These databases sometimes differ in the number of reactions assigned to a gene, due to differences in the predicted substrate specificity of the enzyme.

References:

Weaver, Daniel S., et al. "A genome-scale metabolic flux model of *Escherichia coli* K-12 derived from the EcoCyc database", BMC Systems Biology (2014)

Substrate uptake and growth rate predictions for growth on fructose (data taken from [2]):

**Substrate**	***In vivo* [2]**	***i*CN1361**	**RehMBEL1391 [1]**
Fructose	-2.6	-2.6*	-2.6*
Oxygen	-4.6 +/- 0.3	-4.91	-5.54
Phosphate		-0.22	-6.55
Sulfate		-0.04	-0.05
Ammonium		-2.63	-2.49
Carbon dioxide		5.21	5.88
Protons		2.31	-8.80

Growth rate (1/h)		0.25	0.23

* the substrate uptake rate was fixed in the GSM

In general, *i*CN1361 and RehMBEL1391 show very similar uptake/production flux and growth rate for growth on fructose. The growth rate is slightly lower in RehMBEL1391 and oxygen uptake slightly higher when compared to experimental results. Also, notable is that RehMBEL1391 consumes protons rather than produces protons, which is probably a result of some reactions not being balanced. If we don't allow protons being transported inside the cell, then the growth rate reduces to 0.2 1/h. The phosphate uptake also seems unrealistic as there is no feasible phosphate sink for that amount.

The ^{13}C data was also used to compare the accuracy of the two models at predicting central carbon metabolism flux for growth on fructose. We observed a correlation of 0.88 (r-squared) for *i*CN1361, and a slightly lower correlation of 0.86 (r-squared) for RehMBEL1391.

Comparisons of gene essentiality predictions from *i*CN1361 and RehMBEL1391 [1] to the TraDIS experiments carried out in this manuscript:

	Essential (TraDIS)	Non-essential (TraDIS)
Essential (iCN1361)	127 (TP)	79 (FN)
Non-essential (iCN1361)	29 (FP)	1080 (TN)
Essential (RehMBEL1391)	114 (TP)	116 (FN)
Non-essential (RehMBEL1391)	45 (FP)	1035 (TN)

The above numbers of true positives (TP), false positives (FP), true negatives (TN) and false negatives (FN) results in an overall accuracy, precision and recall of 92%, 81% and 62% for *i*CN1361 and 88%, 72%, 50% for RehMBEL1391, respectively.

Biomass reaction might also need to be updated. It looks like that biomass reaction still contains pyridoxine as the required cofactor instead of pyridoxal-5-phosphate (pydx5p) as corrected by [1].

We thank the reviewer for identifying this correction made in [1] to the biomass equation. We have now updated the biomass reaction in *i*CN1361 to include pydx5p instead of pyridoxine. We have also re-run the model simulations with this update. We found minimal changes to the results. There was a small change to the gene essentiality results due to the gene H16_A2802, which encodes a pyridoxine oxidase (EC: 1.4.3.5), changing from being non-

essential to essential in the GSM. This gene was classified as essential in the TraDIS data, and thus we now have 1 less false negative. Re-running the iMAT algorithm resulted in slightly different condition-specific models (*i*CN1361-f16 and *i*CN1361-f26), which subsequently resulted in some minor changes to the statistics provided in the manuscript. We have summarised these changes in the table below:

Statistical value in the manuscript	Original manuscript	Revised manuscript
Number of up-regulated reactions (line 687)	63	66
Number of down-regulated reactions (line 688)	389	396
Number of biomass-related reactions (line 691)	383	390
Correlation of RuBiSco reaction to TCA reactions (line 715)	0.75	0.76
Correlation of RuBiSco reaction to pyruvate dehydrogenase (line 715)	0.68	0.8
Number of reactions predicted as post-translational controlled (line 769)	145 (32%)	143 (31%)

Importantly, none of the conclusions in the results and discussion have changed after making this update despite these small variations to the statistical values.

Please note that due to these corrections we now submit an updated version of the Supplementary Files 1 – 5 and Figure 4.

The latest RehMBEL1391 study [1] performed gene essentiality experiments but used a different experimental approach (Tn-seq). It also included gene essentiality data from formate/succinate carbon source experiments. It is recommended to include these datasets to test the GSM predictions as well. Since the experimental approach to determine gene essentiality is different, any limitations of TraDIS mentioned by the authors in pages 17-19 did not shown up in the Tn-seq study?

Comparing the results from the GSM and the TraDIS data with a third set of predictions would be a great improvement to the paper, particularly for the cases where the two approaches disagreed, so we would like to thank the reviewer for this suggestion. Firstly, we carried out a comparison between our TraDIS results and the results from Tn-Seq data. Both approaches use transposon insertion sequencing to determine the fitness of genes and therefore we would expect similar results. By comparing the two approaches, we found that both classified 338 genes as essential and 5262 genes as non-essential for growth on fructose minimal media (FMM). Quite surprising, however, is the number of additional non-intersecting genes that each approach found (TraDIS found 237 essential genes that were non-essential in Tn-Seq

and, similarly, the Tn-Seq approach found an additional 234 genes that TraDIS classified as non-essential). The reason for these many discrepancies may be due to the small number of mutants used in the Tn-Seq library (1 M in the TraDIS approach compared to 60,000 in the Tn-Seq analysis – please see the Table below), which may increase the probability of misclassifying non-essential genes as essential. Additionally, we expect that the differences in experimental setup and data analysis methods used for classifying genes, likely contributed to a small number of discrepancies. Furthermore, classification of genes that have both essential and non-essential domains may also differ between the two *in vivo* analyses. These essential genes may have high overall insertion indices, and are thus problematic to classify using approaches such as TraDIS and Tn-Seq. A more complex analysis would be required to accurately classify these genes but is beyond the scope of this project and left for future work, particularly since our main purpose was to use the data to validate the GSM. We provide the TraDIS results for 6637 *C. necator* H16 genes on the GitHub repository (https://github.com/SBRCNottingham/CnecatorGSM/blob/main/JupyterNotebooks/Data/TraDIS_results.xlsx). This file includes the $\log_2$(IPKM+1) index, together with our classification and the Tn-Seq classification, so that readers can assess the likelihood of a gene being essential or non-essential. Readers that are interested in the essentiality of genes that have a $\log_2$(IPKM+1) that is close to the thresholds (as defined in the revised manuscript), particularly if they also disagree with the Tn-Seq data, may want to manually analyse the TraDIS data, as was done in [3]. We have updated the manuscript to include the differences in coverage between the two approaches, please see the new Table 2 and line 393 – line 404 in the results section, in which we conclude that the larger number of transposon insertions achieved in our study provides a more complete coverage of the *C. necator* H16 genome. Because of this, we believe that our analysis can more confidently assess gene essentiality, due to the reduced number of falsely classified essential genes (or false negatives when comparing to the GSM) that is likely to result from a transposon mutant library with significantly lower coverage (i.e., fewer insertions results in higher chance of a gene being missed and falsely classified as essential). A comparison of the number of discrepancies between the two approaches (as described above) is included in the Supplementary Jupyter Notebook ('GSMCnecator_Validation.ipynb') on the GitHub repository (please also see a figure below summarising the comparison). We also provide average transposon insertion frequencies data for each of the three replicons of *C. necator* H16 (chromosomes 1 and 2 and the pHG1 megaplasmids) for our TraDIS libraries grown in SOB and FMM in Tables S12 and S13.

Despite the low coverage of the Tn-Seq analysis, we found that the GSM performance scores are relatively similar when using either approach, but with a lower precision and recall score when using the Tn-Seq predictions (88% overall accuracy, 77% precision and 51% recall). Furthermore, the Tn-Seq data provides a useful source for further analysis of the false positives and false negatives identified between the GSM and TraDIS analysis, as suggested by the Reviewer. Out of the 29 false positives that we observed, the Tn-Seq classifications for 19 genes agreed with the TraDIS data, whereas only 9 agreed with the GSM (note that 1 gene was undefined in Tn-Seq). Similarly, out of the 79 genes identified as false negatives in the

TraDIS vs. *i*CN1361 comparison, 55 agreed with the TraDIS data, whereas 21 agreed with the GSM predictions (and 3 genes were undefined). Included in the 55 genes that the Tn-Seq classifications agreed with our TraDIS classifications, were most of the genes being discussed on pages 17 – 19. This includes all the genes of the NADH dehydrogenase (except for H16_A1060 encoding for the subunit *nuoK*), the succinate dehydrogenase genes, 2-phospho-D-glycerate hydrolase gene and the genes corresponding to the 2-oxoglutarate dehydrogenase complex, which further supports the idea that alternative solutions in the GSM are infeasible *in vivo* due to regulatory constraints. The TraDIS and Tn-Seq approaches, however, are very similar and thus are likely to have the same (or comparable) limitations, and thus it is not a completely independent approach to use as a third comparison. We have updated the results section pages 20 – 26 to include the comparisons with the Tn-Seq classifications.

As the reviewer pointed out, the authors of [1] also include Tn-Seq analysis for growth on formate and succinate, which is useful for further validation of the GSM. We have therefore predicted gene essentiality in *i*CN1361 for both substrates and provide the results in an additional Jupyter Notebook on our GitHub repository ('GSMvalidation_TnSeqEssentiality.ipynb'). For growth on formate, the model achieved an overall score of 89%, a precision score of 71% and a recall score of 52%, whereas for growth on succinate the model achieved an overall accuracy of 89%, a precision score of 71% and a recall score of 50%. Note that these results do not fit in the main manuscript and therefore are only provided as supplementary material on our GitHub repository (https://github.com/SBRCNottingham/CnecatorGSM/blob/main/JupyterNotebooks/GSMvalidation_TnSeqEssentiality.ipynb).

Please note that we have also improved our TraDIS classification approach in the revised manuscript. Firstly, we have reassessed the TraDIS data for areas of low coverage, which allowed us to validate an additional 113 genes in the GSM. Secondly, some gene annotations, which had previously resulted in a mismatch with the GSM and, thus, were excluded from analysis, have been corrected. Thirdly, we have classified genes as essential and non-essential in the TraDIS data using a slightly modified criterion. Previously we used the 75th percentile of the $\log_2(IPKM+1)$ values of genes with known essentiality phenotype to determine whether a gene was essential or non-essential (i.e., $\log_2(IPKM+1) < 75^{th}$ percentile is essential and $\log_2(IPKM+1) > 75^{th}$ percentile was non-essential). Genes that have a $\log_2(IPKM+1)$ value that lies close to the cut-off, however, likely results in potentially incorrect false positives and false negatives. A gene that includes both essential and non-essential domains is likely to have an IPKM value that falls into this region, as described in [3], and briefly above. As previously mentioned, a more complex method is required to reliably classify such genes and remains a challenge for future work. In our revised manuscript, however, we instead define a region of uncertainty (ranging from the 65th percentile and 75th percentile), where the overlap between essential and non-essential insertion numbers is likely to be significant. A total of 39 genes in *i*CN1361 had insertion indexes included in this region of uncertainty and were thus not

classified as either essential or non-essential in our analysis. Similar approaches have been used in previously published transposon sequencing studies, including the Tn-Seq analysis carried out in *C. necator* H16, where 220 genes (33 of which were in *i*CN1361) were not classified. Modifying the criteria for classification made minimal changes to the comparisons to the GSM. However, the total number of genes classified as essential based on TraDIS data for fructose minimal media did decrease significantly from 960 to 608, which is comparable to the number of essential genes identified in other bacterial species using similar approaches. The manuscript has been updated from line 381 – line 392 of the results section and line 1360 – line 1365 in the methods section (please also see below) using the new criteria for classification. The confusion matrix using the updated approach is also shown below. Please also find an updated SupplementaryFile4_GeneEssentiality.xlsx (Table S9) that now also includes the Tn-Seq essentiality phenotypes, defined in [1].

The main changes to the manuscript are provided below. Please see the marked-up manuscript to see all minor changes to the gene essentiality section.

Line 381 – line 392 (results):

A log_2(IPKM+1) (insertion index) threshold of 3.9 was determined as described in the Methods section, so that the *C. necator* H16 genes with insertion indexes equal or lower than this cut-off value (log_2(IPKM+1) ≤ 3.9) were identified as essential (see Supplementary File 4 - Table S8 & Methods for details). Similarly, a log_2(IPKM+1) threshold of 4.4 was determined, such that *C. necator* H16 genes with an insertion index greater or equal to this cut off (log_2(IPKM+1 ≥ 4.4) were classified as non-essential. Genes with an insertion index falling in between the 3.9 and 4.4 thresholds were not classified as either essential or non-essential. Also note that, due to low sequencing coverage of some genomic regions of *C. necator* H16, a total of 204 and 198 genes were excluded from the TraDIS analysis for the SOB and FMM conditions, respectively (see Supplementary File 4 – Table S8 for details). Notably, however, only 20 and 15 of these genes are involved in the GSM, respectively.

Line 393 - line 404 (results):

Gene essentiality estimations have recently been published for *C. necator* H16 grown on fructose, as well as on other carbon sources (25), using Tn-Seq analysis. Notably, however, our TraDIS approach uses a significantly larger transposon mutant library (over 1 M mutants compared to approximately 60,000 mutants in the Tn-Seq analysis – see Table 2 for a detailed comparison of these two datasets), and thus provides a more complete coverage of the genes in terms of insertions. Nevertheless, we have used the data to further validate our gene classifications, particularly for cases where the GSM and TraDIS analysis differed, as discussed in the next section.

Table 2: Comparison of transposon mutant library sizes and average insertion frequencies between TraDIS and the Tn-Seq analysis from (25)

Library	Size of coding regions (bp)	Number of genes analysed	Average gene length (bp)	Total insertions	Insertions after curation	Unique insertions	Average insertions per gene
TraDIS (FMM)	6,454,517	6,637	972.2	9,415,576	7,158,925	1,335,710	201.2
TraDIS (SOB)	6,454,517	6,637	972.2	8,315,651	6,226,973	1,050,800	158.2
Tn-Seq (LB)	6,106,532	6,549	932.4	107,708	84,761	60,000	9.2

Line 414 – line 417 (Results - updated confusion matrix):

Table 3: Confusion matrix showing the number of true positives (TP), true negatives (TN), false positives (FP) and false negatives (FN), obtained by comparing TraDIS results with GSM predictions.

	Essential in GSM	Non-essential in GSM
Essential in TraDIS experiments	127 (TP)	79 (FN)
Non-essential in TraDIS experiments	29 (FP)	1080 (TN)

Line 429 – line 432 (Results):

For the sake of comparison, we also tested the accuracy of *i*CN1361 using the Tn-Seq data and found a similar result for the overall accuracy (88%) and precision (77%) scores but a weaker recall score (51%).

Line 869 – line 881 (Discussion):

Comparing the discrepancies observed in our analysis to the Tn-Seq gene essentiality predictions available from a recent study (25), showed that 70% of the false negatives agreed with the TraDIS results, further suggesting additional constraints are required to correct these gene KO phenotypes in *i*CN1361.

To address the discrepancies between the GSM and TraDIS gene essentiality predictions defined as false positives, a classical genetics approach was carried out to test the fitness of *C. necator* H16 mutants, carrying the in-frame deletions of 6 genes

that were predicted to be essential by *i*CN1361 but not by TraDIS (or by the Tn-Seq predictions for 3 out of the 6 genes), during growth in FMM. None of these mutants grew under these experimental conditions, thus confirming the *in-silico* predictions. The ambiguity of gene essentiality for genes that lie close to the determined threshold, as well as intrinsic limitations of the TraDIS-based approaches are likely responsible for these misclassified genes.

Line 1360 – line 1365 (methods):

The 65th percentile of the log-distribution of the SOB-specific IPKM values (log_2(IPKMc+1)) was then selected as the threshold to classify the *C. necator* H16 genes as essential (log_2(IPKMc+1) ≤ lower threshold), whereas the 75th percentile was used to classify genes as non-essential (log_2(IPKMc+1) > upper threshold). A gene that had a log_2(IPKMc+1) index within the 65th and 75th percentiles was not classified as essential or non-essential in our analysis.

Table S12: Numbers of transposon insertions and average insertion frequencies observed for each of the three *C. necator* H16 replicons (chromosomes 1 & 2 and the pHG1 megaplasmid) following growth in SOB rich medium

Replicon	Total Insertions	Genic Insertions	Unique Insertions	Insertion Frequency (bp^{-1})
Chromosome 1	7,667,849	5,131,370	893,295	4.536
Chromosome 2	1,488,845	1,030,755	135,201	21.542
pHG1	180,091	124,278	22,304	20.272

Table S13: Numbers of transposon insertions and average insertion frequencies observed for each of the three *C. necator* H16 replicons (chromosomes 1 and 2 and the pHG1 megaplasmid) following growth in fructose mineral medium (FMM).

Replicon	Total Insertions	Genic Insertions	Unique Insertions	Insertion Frequency (bp^{-1})
Chromosome 1	7,759,047	5,244,196	920,630	4.401
Chromosome 2	2,659,362	1,873,773	391,164	7.446
pHG1	160,800	121,520	23,916	18.906

Summary of the TnSeq analysis vs. TraDIS classifications (included in GSMCnecator_Validation.ipynb)

GitHub link: https://github.com/SBRCNottingham/CnecatorGSM/blob/main/JupyterNotebooks/GSMCnecator_Validation.ipynb

	Number of genes			
	**Essential**	**Non-essential**	**Undefined**	**Total**
**TraDIS FMM**	608	5762	267	6637
**TraDIS SOB**	373	5918	346	6637
**Tn-Seq FMM**	577	5752	220	6549
**Tn-Seq LB**	513	5776	260	6549

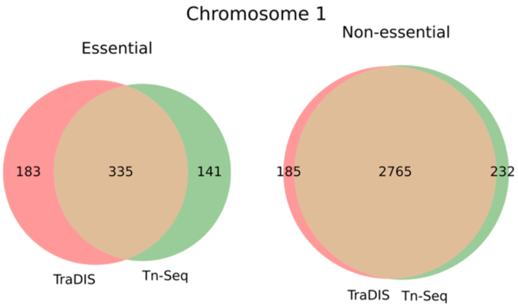

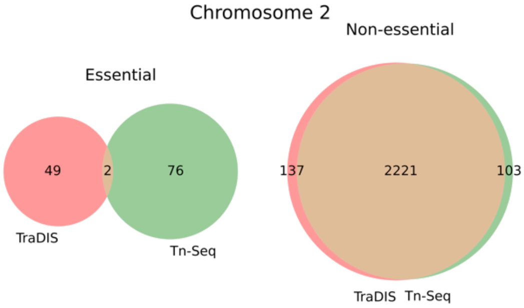

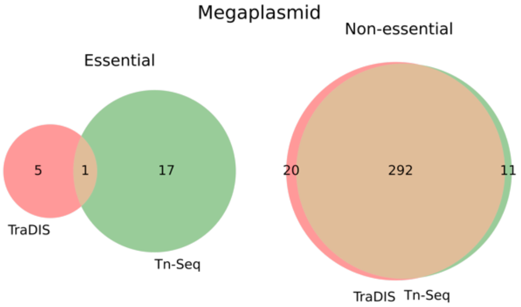

Page 25: iMAT can use transcriptomics data or proteomics data to build condition specific models. Since genome-wide proteomics data is also available in the latest study [1], the authors may want to compare the model they built with proteomic-based iMAT model to find any discrepancies.

We agree that it would be interesting to integrate proteomics data with the model and then compare these results to the transcriptomics-integrated iMAT models. The proteomics data in [1], however, has been collected using continuous culture, whereas the transcriptomics data from [4] that was used in the manuscript are from batch cultures. We were specifically studying the changes in flux and predicting regulation from the exponential growth phase to the PHB-production phase where nitrogen is depleted. Whilst the authors of [1] consider nitrogen-limited conditions, they do this during the growth phase of the bacteria, and thus it is not feasible to make comparisons to the simulations using the transcriptomics data. We therefore do not think it would be suitable to use this data in our manuscript. We do, however, think that integrating the proteomics data from [1] with *i*CN1361, using the iMAT approach, would be interesting as future work to identify flux differences between the four different conditions investigated in their study, and to compare these results to those found using their RBA model. We have added a couple of sentences to the manuscript explaining that

proteomics could be used instead but was not available for the condition we were investigating. We also provide the reference for proteomics data for continuous culture in case of interest to the reader.

Line 919 – line 922 (Discussion):

Notably, the iMAT algorithm can also be integrated with proteomics data, and thus it would be interesting to investigate whether a proteomics-based iMAT model produces similar results. Currently, however, only transcriptomics data are available for *C. necator* H16 growing in batch culture. For continuous culture see (25).

Page 28 lines 627-639: The conclusions the authors made about CBB cycle and CO2 fixation are different from the analysis of [1]. The conclusion from the latest RehMBEL1391 is that "CO2-reassimilation through Rubisco does not provide a fitness benefit for heterotrophic growth, but is rather an investment in readiness for autotrophy". Can the authors provide a more detailed analysis to explain the different predictions made by the two models?

We agree that the conclusions from [1] regarding the CBB cycle should be compared to the conclusions made in our manuscript and therefore would like to thank the reviewer for raising this point. If we have understood correctly, the authors of [1] found a surprising increase in the CBB protein abundance for increasing growth rates when growing on fructose, which they then investigated with their Resource Balance Analysis model. They could not find any fitness benefit of the CBB reactions in terms of biomass or growth rate, and therefore suggest these proteins are under-utilised during fructose growth for 'readiness for autotrophy'. Our analysis, on the other hand, suggests an active CBB cycle is for maximising PHB and/or redox balancing and is based on our results for the nitrogen-limited phase of *C. necator* H16. As part of this analysis, we used iMAT to generate a condition-specific model for the fructose-limited condition and nitrogen-limited condition. The iMAT model representing fructose limitation, did not have the CBB reactions in the model, despite the genes being expressed at a moderate level, in agreement with the results from the RBA model in [1]. The iMAT model representing the nitrogen-limited condition, however, showed an active CBB cycle, in agreement with ^{13}C-tracing experiments that showed CO_2 had been fixed to PHB during nitrogen-limited conditions on fructose. Deactivating RuBisCo in *C. necator* H16, has also been confirmed experimentally to reduce PHB production by 20% in nitrogen-limited conditions. Therefore, whilst the CBB genes are unlikely to provide a fitness advantage during optimal growth, they do appear important for the nitrogen-limited phase. The under-utilisation of CBB proteins during the growth phase may therefore be a readiness for changing to autotrophic behaviour and/or other nutrient-limited conditions, such as nitrogen limitation. We apologise for not making it clear in the original manuscript which conditions we were referring to throughout this analysis, and hence the apparent contradictory conclusions to those reported in [1]. In the results section line 663 – line 666, we define the two iMAT models as *i*CN1361-f16 and *i*CN1361-f26, which correspond to the growth-phase and nitrogen-limited phase (or PHB

phase), respectively, which we agree is not very informative to the reader. We have therefore included '*i*CN1361-f16 (growth phase)' and '*i*CN1361-f26 (nitrogen-limited phase)' to make it clearer to the readers what conditions are being compared throughout these results.

In revisiting this analysis, we have identified a mistake in the CBB flux correlations we have stated in the manuscript. The reactions of the ED pathway had a negative correlation to the CBB reactions, rather than positive as written in the original manuscript. This makes more sense because an active CBB cycle will require some of the flux from fructose-6-phosphate being redirected from the ED pathway and towards the pentose phosphate pathway, as is shown in Figure 5 of the manuscript. We apologise for this mistake and have updated line 711 – line 717 accordingly (please also see below). We have also added some additional analysis with the GSM to confirm that the RuBisCo reaction is beneficial for PHB production fitness. Whilst the flux sampling showed no significant correlation between the CBB genes and PHB production in the iMAT model representing nitrogen-limited conditions, all 10000 flux samples included some flux towards RuBisCo. To test the significance of RuBisCo on PHB yield, we compared the wild-type maximum theoretical yield of PHB to a mutant with RuBisCo deactivated using the original *i*CN1361 model, which confirmed that RuBisCo is important for achieving a maximum PHB yield. We have added an extra sentence to line 717 – line 721 of the results section reporting this result.

Later in the manuscript, on line 800 – line 807 we suggest over-expressing the genes of the CBB cycle during the growth phase of *C. necator*, in order to reduce CO_2 waste on fructose. We have now added an additional sentence on line 807 – line 808 (see also below) explaining that this is not possible unless an additional energy source is provided, in line with the results found in [1].

> **Line 711 – line 721 (Results):**
> From these results, we found an almost perfect anti-correlation to the reactions of the ED pathway ($r < -0.99$), which is due to the flux from glucose-6-phosphate isomerase being redirected towards the pentose phosphate pathway (Fig 5). More interestingly, however, was the high correlation found to reactions in the TCA cycle ($r > 0.76$) and pyruvate dehydrogenase ($r > 0.8$), which supports the idea that the bacteria activate CO_2-reutilising reactions to maximise PHB production (52). Notably, the probability distributions of the CBB reactions were not highly correlated to the PHB pathway reactions. However, by deactivating the RuBisCo reaction from *i*CN1361, whilst maximising for PHB production as the objective, we could show that yield was reduced by 16%, similar to the experimental results from (52).
>
> **Line 807 – line 808 (Results):**
> An additional electron source, such as H_2, however, is likely required to provide the energy to drive the CBB cycle, as also suggested in (25).

In the proteomics data from [1], phaC enzyme level is low compared with phaA or phaB. Is it possible that the feedback inhibition is not the major limitation in f16 condition but the enzyme level of phaC? In other words, is it possible for the computational approach/analysis to be done at the pathway level to make sure that feedback inhibition is the only reason as suggested by the author?

We agree that it would be useful to carry out some additional computational analysis to confirm whether feedback inhibition is the only limiting factor for PHB production during the growth phase. This additional validation, however, would require dynamic modelling of the pathway, which is beyond the scope of this manuscript. We'd like to thank the reviewer however, because re-visiting this section of the manuscript made us realise that our wording was misleading regarding the PHB pathway regulation analysis. We previously stated that post-translational regulation was controlling the PHB biosynthesis pathway flux, and mention the expression levels of *phaA* and *phaB* in particular were extremely high under both conditions and excluded any specific details about *phaC*. The transcriptomics data is in fact in agreement with the proteomics data, such that *phaC* is present during growth on fructose-limitation, but at a much lower level than for the nitrogen-limited condition. The $\log_2$ fold change from fructose-limitation to the nitrogen-limited condition is 1.8, which is considerably higher than the $\log_2$ fold changes for *phaA* ($\log_2$ FC = 0.7) and *phaB* ($\log_2$ FC = -0.6). For this reason, we have reworded this section (line 788 – line 792, also see below) to make it clear that the expression of *phaC* may also be limiting the flux through the PHB pathway during fructose-limited conditions, as the reviewer suggested. We have also added an additional sentence to the discussion (line 927 – line 929, please also see below) suggesting that dynamical modelling would be useful as future work to confirm the proposed regulation, which would be particularly interesting for the conclusions about the CBB cycle and PHB pathway.

Line 788 – line 792 (Results):

Notably, however, *phaC* was only moderately expressed during the growth phase, and subsequently increased in the nitrogen-limited phase ($\log_2$ fold change = 1.8) suggesting that PHA synthase may also be a limiting factor. Over-expression of the *phaC* gene may thus be a suitable target for increasing PHAs.

Line 927 – line 929 (Discussion):

As future work, it would be interesting to carry out dynamic modelling at the pathway level, to validate any candidates proposed from our analysis.

Page 8 lines 184-185: It is great that the authors are using MEMOTE for validation tests and generated a report in the github repository. However, the github repository is not organized using the memote format. For example, there should be a data folder to store the gene

essentiality, growth, and media data. Moreover, the repository can be used to track model changes since new experimental results are often leading the model improvement [2]. It is therefore recommended that the authors follow the same standard to create a memote generated structure. A good example is https://github.com/maranasgroup/iRhto_memote

We thank the reviewers for this suggestion, as we agree that it makes it a lot easier for readers to continue the work with the model, especially as more experimental data becomes available to add to the tests. We have generated a MEMOTE GitHub repository and added the experimental data for testing growth on the various carbon sources and gene essentiality phenotypes for fructose (TraDIS and Tn-Seq data), succinate (Tn-Seq) and formate (Tn-Seq). The new MEMOTE GitHub repository can be found via: https://github.com/SBRCNottingham/CnecatorGSM/tree/main/MEMOTE_repo/sbrc_cnecator_gsm

Minor comments

Page 6 lines 135 - 148: It is possible that some metabolites/reactions in C. necator H16 do not exist in BioCyc. Therefore, it might make more sense to use a more general identifier (e.g., metanetx ids). This will allow the model to use information from other comprehensive databases (e.g., KEGG or Rhea) that are not added into biocyc. Can the authors comment on whether they have encountered this scenario during their model reconstruction?

We agree with the reviewer that different databases include/exclude some metabolites and reactions, which may therefore limit approaches that are derived from one database, as is the case in our construction pipeline, where reactions are extracted from the BioCyc database using ScrumPy software and the BioCyc flatfiles. During our construction, we added some reactions manually due to them being missing, but the function being known experimentally to take place (e.g., the fluorobenzoate degradation pathway). This only happened for a few reactions (~40 reactions), and in these cases we provide the database ID (usually KEGG), for where the reactions were derived in the reaction annotation of the SBML file. Whilst we agree that IDs such as MetaNetX may provide a more useful way of integrating information from different databases, our SBML model is derived from the ScrumPy version of the model, which is generated using BioCyc IDs. Easy updates to the ScrumPy model can be made as the BioCyc flatfiles are updated, which rely on using BioCyc IDs. When using the model, we also find it easier having the BioCyc IDs as the main ID in the model, so that we can easily ‘look up’ reactions and metabolites in the BioCyc database.

Page 44 formulation (3): the objective function should be minimizing of 1-norm of flux vector.

Thank you for identifying this mistake. We have corrected this in the revised manuscript.

There are still 25 unbounded reactions based on memote report, can the authors verify that they are not forming thermodynamically infeasible cycles?

In our MEMOTE report, the 'erroneous energy generating cycles' tests were skipped due to problems with identifying the correct metabolites. We instead tested for energy generation via these metabolites using the Python scripts using MEMOTE locally, which can be found in the supplementary Jupyter Notebook 'Comparisons_RehMBEL1391.ipynb' (https://github.com/SBRCNottingham/CnecatorGSM/blob/main/JupyterNotebooks/Comparisons_RehMBEL1391.ipynb). In this Jupyter Notebook we confirm that both *i*CN1361 and RehMBEL1391 do not include any thermodynamically infeasible cycles that result in energy generation.

Reviewer #2:

Minor comments

When analyzing the correlation between different pathways with the integration of transcriptomic data (e.g., RuBisCo, ED pathway, TCA cycle and PHB). Are those correlations truly the results of integrating the transcriptomic data or are they possibly just the network properties of the model? In other words, are the correlations significant compared to the base model not with transcriptomic data integrated? Comparing this might give a clearer picture.

We agree with the reviewer that it is not clear whether the correlations reported are due to the structure of the network or the integration of the transcriptomics data and have thus revisited this analysis. Notably, when we run flux sampling using the base model, there is a large set of reactions (505) that have not converged (i.e., the flux distribution of these reactions is significantly different when comparing multiple sampling runs). Consequently, neither mean flux values nor correlations can be calculated with high confidence. Importantly, the RuBisCo reaction, the TCA cycle and the pyruvate dehydrogenase reaction all fall into this category. In contrast, the iMAT models contain only three such reactions, which we subsequently removed from any further analysis, as described in our Methods section (line 1215 – line 1218).

However, for the sake of comparison, we have calculated mean flux values and correlations for the base model despite their low confidence levels. Interestingly, we found that the CBB cycle was up-regulated in the nitrogen-limited base model when compared to the growth-phase base model with a mean flux of 0.31 mmol/gDCW/h and 0.06

mmol/gDCW/h, respectively. The correlation of the RuBisCo reaction to the TCA cycle and pyruvate dehydrogenase are significantly smaller (0.24 and 0.32, respectively) when using the base model in comparison to the iMAT model. Note that we mistakenly reported the ED pathway as positively correlated to the RuBisCo reaction, when in fact it was negatively correlated (see also our above response to Reviewer 1). This makes sense since the CBB cycle competes for flux from the fructose-6-phosphate branch point. It is therefore not surprising that we also see a high correlation of the RuBisCo reaction to the ED pathway in the base model. It is, thus, a result of the network structure.

Since many of the results from the base model lack confidence because of the above-described reasons, we feel they should not be added to main manuscript. Instead, we revised the Results section and now describe the problems with the base model and illustrate the importance of additional data to constrain the model further (please see below).

Line 613 – line 629 (Results):

Applying standard FBA however, whilst mimicking nitrogen-limited conditions, resulted in pyruvate and lactate production rather than PHB. Notably, pyruvate production is in-line with experimentally observed behaviour of PHB-negative strains (45). By applying flux variability analysis (FVA), we found that several alternative solutions are available in the model under nitrogen-limited conditions, which can result in various by-products, including acetate, lactate, pyruvate, succinate, formate, ethanol, hydrogen, propionate and also PHB (Fig 3). We next applied flux sampling analysis to *i*CN1361, which generates a probability distribution for each reaction and thus allows us to explore the likeliness that each product is synthesised (46). Using this approach, however, we found that the probability distribution of 505 reactions were significantly different across repeated sampling runs, and thus suggests a lack of convergence due to the high number of alternative solutions. To reduce the number of alternative solutions, and hence the variety of different by-products, we therefore integrated RNA-Seq data derived from nitrogen-depleted conditions to generate a condition-specific version of *i*CN1361, which is highly constrained according to the expression levels of the corresponding genes. We will refer to *i*CN1361, without RNA-Seq integration, as the base model from this point forward to distinguish it from the condition-specific models.

Line 669 – line 675 (Results):

Next, using the two condition-specific models we investigated the differences in metabolic fluxes between the two conditions. For this analysis, we again applied flux sampling to each model to explore the possible range of feasible flux values for each reaction. Unlike the sampling results of the *i*CN1361 base model, only 3 reactions in the iMAT models were not able to converge in the sampling results. The probability

distribution for each reaction was subsequently used to assess whether the flux had significantly altered between the two conditions.

During the revision initiated by Reviewer 2, we realised that our previous statement that the iMAT model was able to detect an active CBB cycle, whereas standard FBA did not, is somewhat misleading. As discussed above, we found solutions with an active CBB cycle using standard FBA but they are not converging into a reliable distribution. However, whilst the CBB cycle was active in both sampling results, the product spectrum was still significantly different, which was the initial reason for the integration of transcriptomics data. Indeed, the mean PHB production flux is only 0.06 mmol/gDCW/h in the base model, whereas it is around 2 mmol/gDCW/h in the iMAT model. Despite the lack of confidence in the base model results, we have still removed the statement in the discussion that claimed the iMAT model was the only model that had an active CBB cycle, since it no longer holds, and we instead emphasise the need to integrate the data to remove the problem of convergence.

Line 914 – line 919 (Discussion):

Importantly, an active CBB cycle for growth on fructose has previously been observed experimentally but could not be predicted using the *i*CN1361 base model, due to the high level of redundancy in the model that prevent the results from converging in our analysis. Both results demonstrate the importance of solution space reduction by experimental data for a reliable analysis of biological properties using GSMs.

Is the manually curated PGDB in BioCyc format available online? This will help a lot to share the results especially to non-computational people.

Unfortunately, we do not have an online version of the BioCyc PGDB available, however we are currently trying to generate a curated PGDB of *i*CN1361, which we hope to have available upon publication on our GitHub repository. The readers will be able to use these files to create a local database using the PathwayTools software, where they will be able to visualise the metabolic reactions (in the same way you can on the BioCyc website) and perform flux balance analysis within the software (again in the same way you can with the online version). We think that the availability of the model to a more user-friendly platform, such as the PathwayTools software, which does not require programming skills, will make the model usable to a broader group of people, and therefore we wish to thank the reviewer for this suggestion.

References

[1] Jahn, Michael, et al. "Protein allocation and utilization in the versatile chemolithoautotroph *Cupriavidus necator*", eLife (2021).

[2] Park, Jong M., Genome-scale reconstruction and in silico analysis of the Ralstonia eutropha H16 for polyhydroxyalkanoate synthesis, lithoautotrophic growth, and 2-methyl citric acid production. BMC Systems Biology (2011)

[3] Goodall, Emily C. A., et al. "The Essential Genome of *Escherichia coli* K-12", mBio (2018)

[4] Shimizu, Rie, et al. "Detection of phase-dependent transcriptomic changes and Rubisco-mediated CO_2 fixation into poly (3-hydroxybutyrate) under heterotrophic condition in *Ralstonia eutropha* H16 based on RNA-seq and gene deletion analyses", BMC Microbiology (2013)

Submitted files:

- Marked-up manuscript (includes a new Figure 5 due to previous version being slightly cut at the side and an updated Figure 4 due to slight change in result from the update to the biomass equation)
- Clean manuscript
- All 6 Supplementary Files (due to re-running all the analysis after updating the biomass equation and gene essentiality cut-off value)

Updates to the GitHub Repository:

- Updated GSMCnecator_Validation.ipynb (includes comparisons to Tn-Seq data)
- Updated GSMCnecator_NitrogenLimitation.ipynb (now includes flux sampling of the base *i*CN1361 model)
- New Jupyter Notebook Comparisons_RehMBEL1361.ipynb (network property comparisons and comparisons of model simulations)
- New Jupyter Notebook GSMvalidation_TnSeqEssentiality.ipynb (Assessment of *i*CN1361 using the TnSeq data for fructose, formate and succinate).
- New figure showing the ScrumPy model construction pipeline to guide readers in both understanding the model and for further development.
- Updated 'TraDIS_results.xlsx' for improved annotation and modified cut-off determination
